# Bismuth fire assay preconcentration and empirical coefficient LA-ICP-MS for the determination of ultra-trace Pt and Pd in geochemical samples

**DOI:** 10.1038/s41598-022-15881-5

**Published:** 2022-07-07

**Authors:** Wenshan Ni, Xiangju Mao, Mingxing Yao, Xiaorui Guo, Qiliang Sun, Xiaofei Gao, Hongli Zhang

**Affiliations:** 1 Zhengzhou Institute of Multipurpose Utilization of Mineral Resources, CAGS, 328 Longhai West Road, Zhengzhou, 450006 People’s Republic of China; 2 China National Engineering Research Center for Utilization of Industrial Minerals, Zhengzhou, 450006 People’s Republic of China; 3 Key Laboratory of Evaluation and Multipurpose Utilization of Polymetallic Ores Ministry of Natural Resources, Zhengzhou, 450006 People’s Republic of China

**Keywords:** Solid Earth sciences, Chemistry

## Abstract

In this work, a novel method of solid sample pretreatment technique of bismuth fire assay (Bi-FA) combined with solid sample determination by laser ablation ICP-MS (LA-ICP-MS) was reported for the determination of ultra-trace Pt and Pd in geochemical samples. Bismuth oxide (Bi_2_O_3_) was used as fire assay collector to directly enrich Pt and Pd from solid samples, and Ag protection cupellation was employed to generate Ag granules. After cleaning, weighing and annealing, the Ag granules were compressed into thin slices and determined by LA-ICP-MS for ^195^Pt, ^105^Pd and ^109^Ag (^109^Ag was selected as the internal standard isotope). Bi_2_O_3_ provided exceptionally low blanks compared to nickel oxide and lead oxide commonly employed in fire assay procedures, and could be applied directly without purification. Different from traditional empirical coefficient method, the Chinese Certified Reference Materials (CRMs) for Pt and Pd were treated by the same procedure to obtain completely matrix matched Ag slices. And then modified empirical coefficient method and internal standard calibration strategy was used to reduce the instability of LA-ICP-MS, and random multipoint laser ablation was employed to further reduce analytical variation resulting from heterogeneity of Pt and Pd in the Ag slice. Under optimal conditions, excellent calibration curves for Pt and Pd were obtained (0.407–2958 μg g^−1^ and 0.407–2636 μg g^−1^, respectively), with correlation coefficients exceeding 0.9996. The method detection limits for Pt and Pd were 0.074 and 0.037 ng g^−1^, respectively. The established method was applied successfully to analysis of real geochemical samples, with determined values in good agreement with the results of traditional Pb-FA graphite furnace atomic absorption spectrometry (GF-AAS), and spiked recoveries between 87.8 and 125.0%.

## Introduction

Laser ablation (LA) due its capability of complete ablation of any solid material, is the most frequent universal sample introduction technique for solid samples^1,2^. Laser ablation inductively coupled plasma mass spectrometry (LA-ICP-MS) has become a powerful analytical tool for sensitive ultra-trace analysis of solid samples in recent years in different application fields, such as geological samples^3,4^, metallic and semiconducting materials, environmental and biological samples^5–7^ and so on.

Pt and Pd are known for their valuable properties and rare resources, which have been widely applied in petroleum, automobile, electronics, chemical industry, atomic energy, as well as environmental protection industry^8–10^. Consequently, accurate determination of Pt and Pd in geochemical samples is of great significance for geological science research and precious metal ore prospecting. However, it is difficult to accurate determination of ultra-trace Pt and Pd because of the low abundance and uneven distribution in natural Pt–Pd ore as well as the nugget effect^11,12^. Therefore, appropriate sample pretreatment techniques of Pt and Pd were needed prior to the determination.

Fire assay is an ancient but still used method, which plays an important role in the separation and enrichment of precious metals. The solid geochemical samples were mixed and reacted with solid fluxing agent at high temperature, then the target precious metals were concentrated by collectors to product high density alloy granule, conversely, the nonprecious metals and rock-forming elements reacted with solid flux to produce low density silicate or borate fluids^13–16^. Thereby, the target precious metals were successfully separated from the sample matrix. Based on the difference of collectors, fire assay methods can be divided into nickel sulfide fire assay (NiS-FA)^16,17^, lead fire assay (Pb-FA)^18,19^, antimony fire assay (Sb-FA)^20^, tin fire assay (Sn-FA)^21,22^ and so on. NiS-FA and Pb-FA are the most commonly used methods to simultaneously concentrate Pt and Pd in geochemical samples. In previous publications, also Pb fire assay buttons^23–25^ and NiS buttons^26,27^ were determined by LA-ICP-MS for precious metal when using external calibration against matrix-matched standards. However, due to the high and changeable reagent blank mainly from the NiO and PbO collector the accurate determination of ultra-trace Pt and Pd has become very difficult, thus the collector reagents must be purified in advance^17,19^. The selectivity of Sb-FA was unsatisfactory^20^ and Sn granules could not be removed by cupellation^22^. Therefore, other novel fire assay collectors were constantly searching and trying. Pt and Pd could form a series of alloys or metal inter-compounds with non-toxic Bi at high temperatures; thus Bi could quantitatively collect the precious metal elements in solid geological samples^28^.

In this work, a novel method of Bi-FA preconcentration combined with empirical coefficient method LA-ICP-MS for the determination of ultra-trace Pt and Pd in geochemical samples was established. Non-toxic Bi_2_O_3_ was used as the fire assay collector to enrich the precious metal elements into Bi granule, and Ag protection cupellation was employed to form Ag slice for direct laser ablation solid sample injection. Compared with Pb-FA and NiS-FA, the reagent blank of Bi_2_O_3_ was relatively low. Thus Bi_2_O_3_ could be directly employed to collect precious metal elements without purifying. Moreover, the harm of toxic collector to the analyst and environment was avoided by using the non-toxic Bi_2_O_3_. The Chinese Certified Reference Materials (CRMs) of Pt and Pd were treated by the same procedure to obtain completely matrix matched Ag slices, and the modified empirical coefficient method was employed to fit the correction curve. Laser ablation analysis of the Ag slice for direct solid sample injection was used to avoid acid digestion that was used in the traditional GF-AAS or ICP-MS determination methods, which saved the analysis time, reduced the blank value, decreased the interference of polyatomic molecular ions and the dilution effect, and eliminated acid reagents to protect the environment and the health of the analyst. The established method was successfully applied to determine Pt and Pd in real geochemical samples, and the determined values were in good agreement with the results of traditional Pb-FA GF-AAS method.

## Experimental details

### Instrumentation

A laser ablation system (Model GeoLas HD, Coherent, USA) coupled to the quadrupole (Q) ICP-MS (Model 7700x, Agilent, USA) was used in all experiments for the determination of Pt and Pd. The operating parameters laser ablation and mass spectrometric measurements are summarized in Tables 1 and 2. Millionth electronic balance (ME5, Sartorius, Germany) and micropipettors (100–1000 μL, Brand, Germany) were used for weighing and pipetting.

**Table 1:** Laser ablation system operating condition.

Laser ablation system	ArF excimer laser
Type specification	GeoLas HD
Wavelength	193 nm
Energy density	7 J cm^−2^
Repetition rate	6 Hz
Spot size	60 μm
Pulse number	200
Carrier gas (He) flow rate	0.6 L min^−1^

**Table 2:** ICP-MS operation conditions.

ICP-MS instrument	Agilent 7700x
RF power	1550 W
Auxiliary gas (Ar) flow rate	1.00 L min^−1^
Plasma gas (Ar) flow rate	15 L min^−1^
Sampling depth	7.5 mm
Measurement mode	STD
Survey runs	jump
Measured isotopes and dwell time per isotope	20 ms (^195^Pt, ^105^Pd) 10 ms (^109^Ag)
No. of sweeps	1
Detector mode	Dual

### Standard solutions and reagents

The mixed standard solutions (Pd and Pt 10 μg mL^−1^) in 10% aqua regia bought from SPEX CertiPrep group (USA). Ag standard solution (25 g L^−1^) was prepared and used as cupellation protector.

The fire assay collector of Bi_2_O_3_ and other solid fluxing agent such as Na_2_CO_3_, Na_2_B_4_O_7_·10H_2_O, Na_2_O_2_, CaO, SiO_2_ and flour were of analytical reagent grade (AR) and purchased from Tianjin Kemiou Chemical Reagent Co., Ltd. HCl and HNO_3_ were of excellent reagent grade (GR, Kemiou Chemical Reagent Co., Ltd, Tianjin). Ultrapure water (18.2 MΩ cm) obtained from a Milli-Q water purification system (Millipore, Bedford, MA, USA) was used throughout the whole experiments.

### Sample pretreatment and determination

#### Fire assay recipes, melting process, cupellation and Ag granule pretreatment

According to the mineral composition characteristics of geochemical samples (such as rock, soil, river sediment, chromite, black shale and polymetallic ore), the fire assay recipes were adjusted, as shown in Table 3. In order to have better sample decomposition and enrichment effect, some special samples should be pretreated before fire assay^29^. For example, sulfide rock sample should be heated in a furnace at 650 °C for 2 h; and chromite sample should be mixed well with CaO and Na_2_O_2_ and heated at about 680 °C for 1.5 h.

**Table 3:** Bi fire assay recipes for geochemical samples.

Sample type	Recipe (g)					
	Sample weight	Na_2_B_4_O_7_⋅10H_2_O	Glass powder	Na_2_CO_3_	Bi_2_O_3_	Flour
Silicate rock	10–20	20	20	50	40	4
Carbonate rock	10–20	20	25	50	40	4
Sulfide rock	10–20	20	25	55	40	4.5
Soil	10–20	20	15	50	40	4
Stream sediment	10–20	20	15	50	40	4
Black shale	5	20	20	50	100	3
Polymetallic ore	10	15	20	50	100	5
Chromite	10	25	20	30	50	6
Chinese certified reference materials	10	20	15	50	40	4

Raw materials according to Table 3 were mixed well in an ingredient bottle, 70% of the mixture was transferred into a 500 mL fire-clay crucible, then 250 μL of Ag standard solution (25 g L^−1^) was added. After drying, another 30% of the mixture was added and 20 g of covering agent (Na_2_CO_3_, Na_2_B_4_O_7_·10H_2_O, glass powder, Bi_2_O_3_ and flour were mixed well at 50 g: 20 g: 15 g: 10 g: 4 g) was uniformly added. Then the crucible was fused in a furnace heated to 1070 °C gradually and kept for 30 min. The melts in the crucible were poured into a cast iron mold. Once cooled, the Bi granule was separated from the slag.

The Bi granule was cupellated in a magnesia cupel at 940 °C until it produced a dazzling color and flashes, which was the end of cupellation process. At this stage, Bi in the granule was eliminated and the target precious metal elements were trapped in the Ag granule. The Ag granule was ultrasonic cleaned, weighed and annealed at 700 °C for 30 min, then was compressed into a thin slice (~ 0.2 mm). A blank sample was also subjected to this procedure.

#### Standard samples preparation

According to the fire assay recipes of Chinese Certified Reference Materials (CRMs) in Table 3, 10 g of CRMs (GBW07288, GBW07289, GBW07290, GBW07291, GBW07293, GBW07294, GBW07341 and GBW07342) and solid fluxing agent were mixed well in parallel. Then the next Ag standard solution and covering agent adding procedure and the preparation of external standard CRMs Ag slices steps were the same as “Fire assay recipes, melting process, cupellation and Ag granule pretreatment” section.

### LA-ICP-MS determination

The slices of external standard CRMs series and real geochemical sample were determined by random multipoint (10 points) LA-ICP-MS for ^195^Pt, ^105^Pd and ^109^Ag (internal standard). The mass fractions of Pt and Pd in real geochemical samples were calculated by formula (1) as shown in Ref.^29^:

1 $$w = \frac{{w_{sam} \times m_{sam} - w_{0} \times m_{0} }}{{m_{s} }} \times 10^{3}$$

where ${w}_{0}$ is the mass fractions of Pt and Pd in blank Ag slice (μg g^−1^); ${m}_{0}$ is the mass of blank Ag slice (g); ${w}_{sam}$ is the mass fractions of Pt and Pd in real sample Ag slice (μg g^−1^); ${m}_{sam}$ is the mass of real sample Ag slice (g); ${m}_{s}$ is the mass of real sample (g).

## Result and discussion

### Comparison of different collectors

The regent blanks of Bi_2_O_3_, NiO and PbO were respectively determined by Bi-FA-ICP-MS, NiS-FA ICP-MS and Pb-FA GF-AAS, and the values were shown in Table 4. It could be seen that the reagent blank of Pt and Pd in Bi_2_O_3_ was extremely low compared to commercial PbO and NiO. Therefore, Bi_2_O_3_ could be used as collector directly without purification.

**Table 4:** Total procedure blanks (mean data ± standard deviation, n = 5) for Pt and Pd using commercially available Bi_2_O_3_, NiO and PbO (ng g^−1^).

Element	Bi_2_O_3_	NiO	PbO
Pt	0.54 ± 0.12	56 ± 21	2.6 ± 0.9
Pd	0.52 ± 0.09	75 ± 34	3.5 ± 0.6

### Cupellation temperature

**Figure 1:**
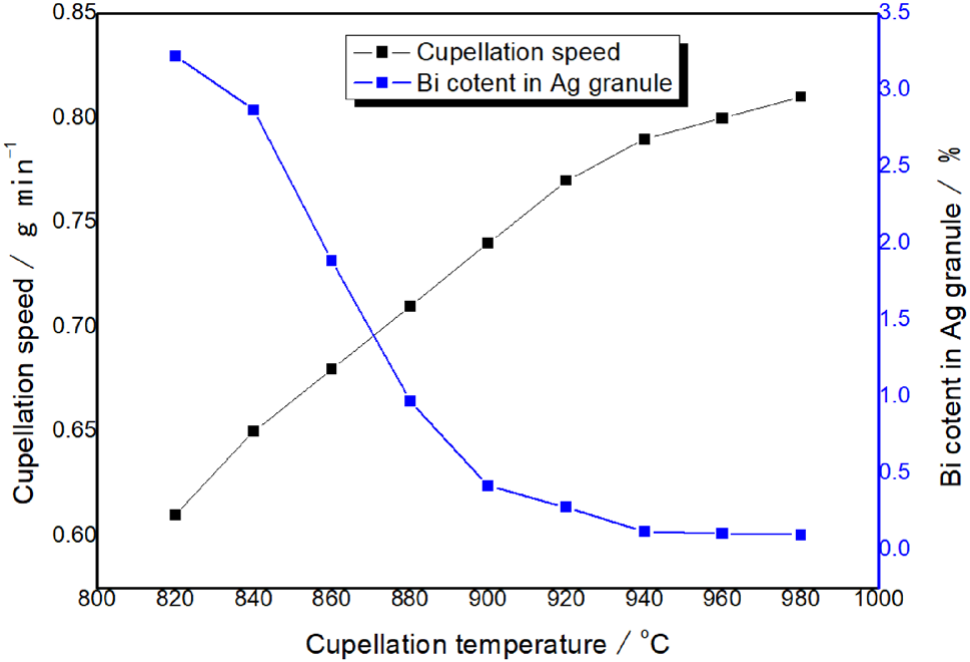
The optimization of cupellation temperature of Bi_2_O_3_.

Metal Bi can be oxidized to Bi_2_O_3_ at ~ 300 °C. However, the melting point of Bi_2_O_3_ is 820 °C. Therefore, the cupellation temperature must be controlled to above 820 °C so that liquid Bi_2_O_3_ could be absorbed by the magnesia cupel. The effect of cupellation temperature was optimized with the data shown in Fig. 1. It can be seen that the cupellation speed was accelerated, and Bi content remaining in the Ag granule was also decreased with increasing of cupellation temperature. When the temperature reached to 950 °C, obvious volatilization of Bi_2_O_3_ could be observed. If the temperature was too high, the loss of Pt and Pd as well as muffle furnace would be increased. Therefore, 940 °C was selected as the cupellation temperature.

After Bi cupellation, the target Pt and Pd were trapped in the Ag granule. The Ag granule was annealed at 700 °C to further homogenize the alloy of Pt, Pd and Ag, and which was compressed into ~ 0.2 mm slices to facilitate the use of the laser ablation system.

### Preparation of Ag slices

**Figure 2:**
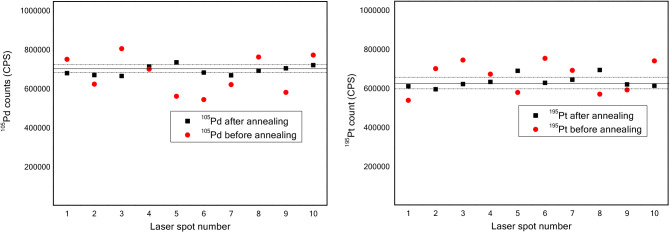
The effect of annealing on signal stability of ^105^Pd and ^195^Pt. Solid line and dotted line were the average and standard deviation after annealing.

The solid samples determined by LA-ICP-MS were required to be as uniform as possible, thus the effect of annealing on the signal stability of ^105^Pd and ^195^Pt were evaluated and shown in Fig. 2. It was observed that, the signal fluctuation before annealing was obviously larger than that after annealing. Therefore, the Ag granules were annealed at 700 °C to ensure the uniformity of the target Pt and Pd inside the Ag slices.

### Mass spectral interferences

Based on the principle of high abundance and free from isobaric interference, the monitored isotopes of Pt, Pd and Ag were shown in Table S1, ^195^Pt and ^105^Pd were selected as measuring isotopes. Though ^107^Ag and ^109^Ag are close in abundance, the mass charge ratio difference of ^109^Ag/^105^Pd is larger than ^107^Ag/^105^Pd, then ^109^Ag was selected as internal standard isotope. The possible interferences from polyatomic molecular ions on ^195^Pt, ^105^Pd and ^109^ Ag were shown in Table S2. After Bi-FA and cupellation, there were only trace Bi, Au, Pt, Pd, Ru, Rh and Ir reserved in Ag granule. Compared to traditional solution injection system, laser ablation solid sample injection could avoid the introduction of large amount of Cl, N, H and O into the ICP. Based on the above means, the possible interferences of polyatomic molecular ions could be effectively decreased.

### Modified empirical coefficient method

The empirical coefficient method is based on the certified values and signal strength of a series of CRMs, using linear or nonlinear regression methods to obtain the coefficients for the quantification formula and allow quantitative sample analysis^30^, which is usually used in X-ray fluorescence analysis of solid samples. However, the traditional empirical coefficient method has a high demand on sample matrix, which requires the composition and structure of the CRMs and the real sample to be tested should be highly similar. Up to now, only a few geochemical Certified Reference Materials included soil, stream sediments, peridotite, chromite and Pt–Pd ores were developed by China. Some special samples, such as polymetallic ore and black shale, the matrix was not identical to the existing CRMs, the accuracy of the method will be affected.

**Figure 3:**
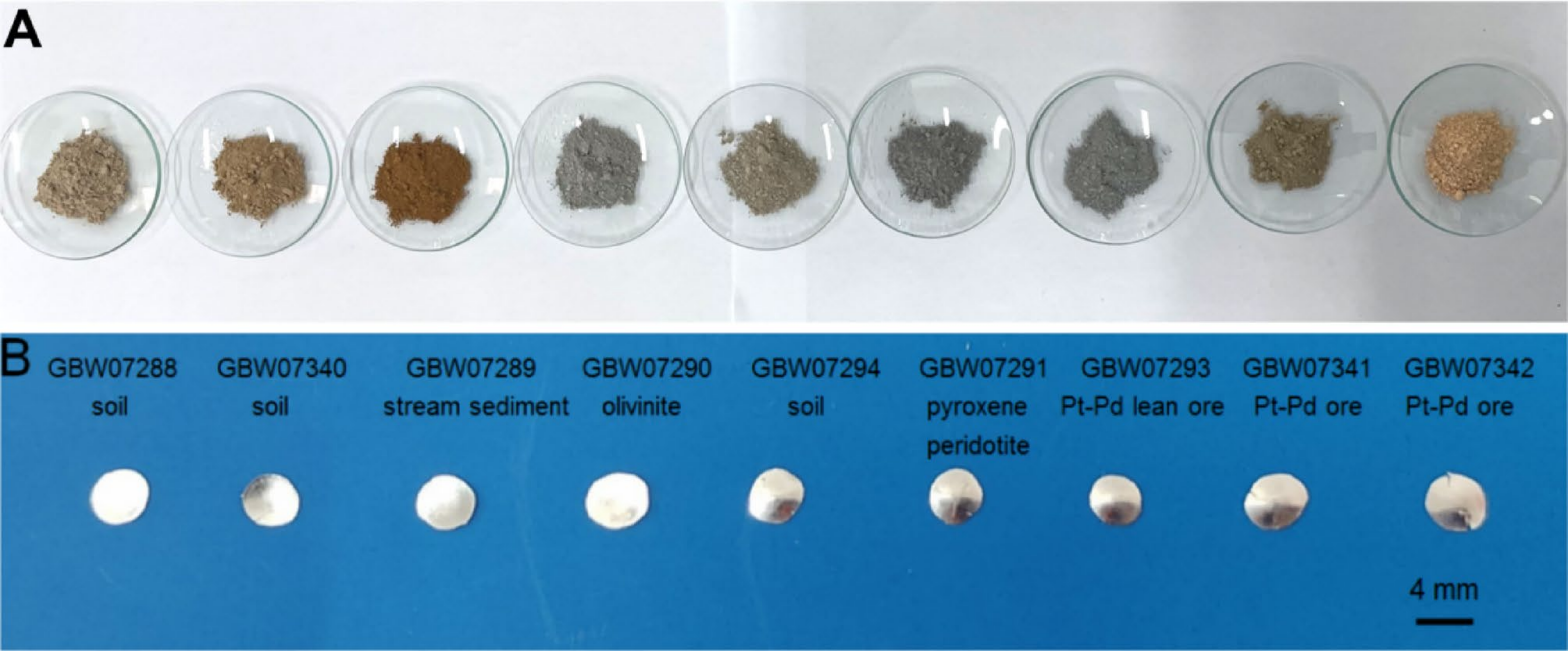
The typical Ag slices of CRMs. (**A**) CRMs before Bi-FA, (**B**) CRMs after Bi-FA.

In this work, a fully matrix-matched Ag slices were obtained and modified empirical coefficient method LA-ICP-MS was established for the determination of ultra-trace Pt and Pd in multiple geochemical samples. Bi-FA was employed to enrich the target precious metal elements from the CRMs and real samples (such as soil, river sediment, chromite, olivinite and Pt–Pd ore) into the Bi granule. After Ag protection cupellation, Pt and Pd were enriched in the Ag slices, fully matrix-matched was achieved and the typical Ag slices of CRMs were shown in Fig. 3. Details of the matrix-matched Pt and Pd mixed external standard CRMs series in the Ag slices are shown in Table 5. The concentrations for Pt and Pd in the media of Ag slices were 0.407–2958 μg g^−1^ and 0.407–2636 μg g^−1^, respectively. The representative time-resolved LA-ICP-MS signals of blank and certified sample were shown in Fig. S1.

**Table 5:** The mass fractions of Pt and Pd in the Ag slices of CRMs.

Standard serials	Certified values of Pt/ng g^−1^	Certified values of Pd/ng g^−1^	m_Ag_/μg	Pt mass fractions in the Ag slices/μg g^−1^	Pd mass fractions in the Ag slices/μg g^−1^
GBW07288	0.26	0.26	6387	0.407	0.407
GBW07340	0.66	0.66	6437	1.025	1.025
GBW07289	1.6	2.3	6292	2.543	3.655
GBW07290	6.4	4.6	6764	9.462	6.801
GBW07294	14.7	15.2	6208	23.68	24.48
GBW07291	58	60	6383	90.87	94.00
GBW07293	440	570	6626	664.1	860.2
GBW07341	1900	570	6423	2958	887.4
GBW07342	–	1670	6335	–	2636

### Internal standard calibration strategy for LA-ICP-MS

In this work, internal standard calibration method was employed to reduce analysis error and correct biases resulting from fluctuations in laser output power as well as sample ablation amount and transport efficiency, to improve the method precision and accuracy. Due to Ag content in the Ag slices were almost identical between CRMs and real samples, then ^109^Ag in the Ag slices was selected as internal standard isotope for the determination of ^195^Pt and ^105^Pd. According to the basic principle and formula (Formula 2) of internal standard method^29^, the concentration of the target element in real sample could be calculated.

2 $$\frac{{w_{t}^{sam} }}{{w_{t}^{std} }} = \frac{{I_{t}^{sam} w_{i}^{sam} /I_{i}^{sam} }}{{I_{t}^{std} w_{i}^{std} /I_{i}^{std} }} = \frac{{I_{t}^{sam} /I_{i}^{sam} }}{{I_{t}^{std} /I_{i}^{std} }}$$

where $w_{t}^{sam}$ and $w_{t}^{std}$ are the concentrations of target element (Pt and Pd) in Ag slices of real and standard samples (μg g^−1^), respectively; $w_{i}^{sam}$ and $w_{i}^{std}$ are concentrations of internal standard element (Ag) in Ag slices of real and standard samples (μg g^−1^), respectively. In our experiment, as the concentrations of internal standard element in real and standard samples were the same by adding the same amount of Ag standard solution during Ag protection cupellation procedure, then the formula is simplified; $I_{t}^{sam}$ and $I_{t}^{std}$ are the signal strength of target element in Ag slices of real and standard samples (cps), respectively; $I_{i}^{sam}$ and $I_{i}^{std}$ are the signal strength of internal standard element in Ag slices of real and standard samples (cps), respectively.

The internal standard and non-internal standard method coefficient of variations (CVs) (n = 10) were compared in Table 6. It is observed that the CVs of ^195^Pt and ^105^Pd by non-internal standard method were between 5.71 and 6.68%. In comparison, the CVs were reduced to 2.54–4.05% when internal standard method was used.

**Table 6:** Determined CVs of non-internal standard and internal standard LA-ICP-MS methods.

	*I*_195Pt_/cps	*I*_105Pd_/cps	*I*_109Ag_/cps	*I*_195Pt_ /*I*_109 Ag_	*I*_105Pd_ /*I*_109 Ag_
1	4.43 × 10^3^	3.52 × 10^3^	5.82 × 10^8^	7.61 × 10^–6^	6.04 × 10^–6^
2	4.77 × 10^3^	3.92 × 10^3^	6.46 × 10^8^	7.38 × 10^–6^	6.07 × 10^–6^
3	4.65 × 10^3^	3.74 × 10^3^	6.16 × 10^8^	7.55 × 10^–6^	6.07 × 10^–6^
4	4.26 × 10^3^	3.56 × 10^3^	5.82 × 10^8^	7.31 × 10^–6^	6.12 × 10^–6^
5	4.29 × 10^3^	3.59 × 10^3^	5.73 × 10^8^	7.48 × 10^–6^	6.26 × 10^–6^
6	4.59 × 10^3^	3.47 × 10^3^	5.82 × 10^8^	7.88 × 10^–6^	5.96 × 10^–6^
7	4.22 × 10^3^	3.53 × 10^3^	5.87 × 10^8^	7.20 × 10^–6^	6.01 × 10^–6^
8	4.29 × 10^3^	3.46 × 10^3^	5.74 × 10^8^	7.47 × 10^–6^	6.04 × 10^–6^
9	4.90 × 10^3^	3.96 × 10^3^	6.17 × 10^8^	7.94 × 10^–6^	6.41 × 10^–6^
10	3.91 × 10^3^	3.31 × 10^3^	5.65 × 10^8^	6.92 × 10^–6^	5.86 × 10^–6^
Average	4.43 × 10^3^	3.61 × 10^3^	5.93 × 10^8^	7.47 × 10^–6^	6.08 × 10^–6^
CVs/%	6.68	5.71	4.29	4.05	2.54

### Analytical performance

The Pt and Pd mixed external standard series were prepared with the concentrations of 0.407–2958 μg g^−1^ and 0.407–2636 μg g^−1^ in the media of Ag slices. The standard series of CRMs are shown in Table 5. At the optimum conditions, the intensities of ^195^Pt, ^105^Pd and ^109^Ag were detected by LA-ICP-MS, and the concentrations of target elements were calculated by formula (2).

The analytical performance of the proposed Bi-FA LA-ICP-MS method has been validated using the calibration curve equation, fit coefficient and LODs, shown in Table 7. Excellent curve fitting of Pt and Pd were obtained and shown in Fig. S2 (0.407–2958 μg g^−1^ and 0.407–2636 μg g^−1^, respectively), with the correlation coefficients exceeding 0.9996. Based on 3δ_blank_ approach as recommended by IUPAC for spectrochemical measurements, the LODs (3 * standard deviation of background/slope of calibration curve, for 10 g sample) of the proposed method for the target Pt and Pd were 0.074 and 0.037 ng g^−1^, respectively. The LODs for Pt and Pd obtained by this method along with other methods were compared. The results in Table 8 revealed that, due to the high enrichment factor (about 1667 fold, 10 g sample weight pre-concentrated into ~ 6 mg Ag granules) the LODs obtained in this work and our previous Pb-FA LA-ICP-MS methods^29^ were much lower than those low enrichment factor methods based LA-ICP-MS and NiS/Pb fire assay^24–27^.

**Table 7:** Analytical performance data by the established Bi-FA LA-ICP-MS method for Pt and Pd.

Isotopes	Linear range/μg g^−1^	Linear equation	R^2^	LOD/ng g^−1^
^195^Pt	0.407–2958	y = 9.59 × 10^-7^x-9.06 × 10^–7^	0.9998	0.074
^105^Pd	0.407–2636	y = 7.35 × 10^-7^x + 4.54 × 10^–6^	0.9996	0.037

**Table 8:** Comparison of detection limits using this proposed technique and other conventional methods.

Analytical technique	LODs		Ref.
	Pt	Pd	
Pb-FA Spark-OES	10 ng g^−1^	100 ng g^−1^	25
Pb-FA LA-ICP-MS	30 ng g^−1^	25 ng g^−1^	
Pb-FA GD-MS	7 ng g^−1^	9 ng g^−1^	
NiS-FA LA-ICP-MS (dynamic reaction cell)	20 ng g^−1^	28 ng g^−1^	26
NiS-FA LA-ICP-MS (focusing sector field MS)	11 ng g^−1^	17 ng g^−1^	27
Pb-FA femtosecond LA-ICP-MS	6 ng g^−1^	9 ng g^−1^	24
Pb-FA LA-ICP-MS	0.06 ng g^−1^	0.03 ng g^−1^	29
Bi-FA LA-ICP-MS	0.074 ng g^−1^	0.037 ng g^−1^	This work

## Sample analysis

Under the optimal experimental and instrumental conditions, real geochemical samples were analyzed by the established Bi-FA LA-ICP-MS method and compared to Pb-FA GF-AAS method. The results are shown in Table 9. It can be seen that the determined values are in good agreement with the results of traditional Pb-FA GF-AAS, and the spiked recoveries were between 87.8 and 125.0%.

**Table 9:** Comparison of analytical data for Pt and Pd in real geochemical samples by the proposed Bi-FA LA-ICP-MS and traditional Pb-FA GF-AAS methods (n = 5, ng g^−1^).

Sample	Bi-FA LA-ICP-MS		Added		Total found		Recovery/%		Pb-FA GF-AAS	
	Pt	Pd	Pt	Pd	Pt	Pd	Pt	Pd	Pt	Pd
1	3.28 ± 0.22	2.71 ± 0.29	5	5	9.53	7.26	125	91	3.06 ± 0.26	2.94 ± 0.32
2	6.72 ± 0.88	9.55 ± 1.12	10	10	15.5	20.6	87.8	111	7.27 ± 1.22	8.80 ± 1.36
3	19.1 ± 2.3	16.7 ± 1.9	20	20	37.6	35.8	92.3	95.6	17.4 ± 2.8	18.0 ± 2.2
4	48.5 ± 4.5	46.5 ± 5.3	50	50	93.3	92.6	89.6	92.2	44.6 ± 5.7	42.3 ± 4.9
5	113 ± 11	123 ± 9	100	100	222	228	109	105	105 ± 12	135 ± 15
6	341 ± 25	306 ± 21	250	250	560	545	87.8	95.6	330 ± 23	325 ± 28
7	805 ± 48	28.1 ± 2.8	1000	50	1875	87.1	107	118	778 ± 43	30.0 ± 3.6
8	84.3 ± 6.6	172 ± 18	100	100	206	278	122	106	80.7 ± 5.8	160 ± 15
9	142 ± 18	23.5 ± 2.8	100	50	257	68.2	115	89.4	151 ± 14	21.3 ± 2.9
10	121 ± 14	405 ± 22	100	500	227	950	106	92.4	124 ± 11	382 ± 28
11	474 ± 41	488 ± 45	500	500	952	1033	95.6	109	455 ± 37	512 ± 39
12	252 ± 24	687 ± 55	500	500	734	1140	96.5	90.7	271 ± 26	652 ± 41
Black shale1	503 ± 45	555 ± 38	500	500	1063	1006	112	90.3	521 ± 32	531 ± 28
Black shale2	231 ± 23	286 ± 28	200	200	408	514	88.7	114	243 ± 22	275 ± 21

## Conclusions

In this work, the method of Ag protection cupellation Bi-FA combined with LA-ICP-MS for the determination of Pt and Pd was established. Bi_2_O_3_ was used as fire assay collector, which has the advantages of lower toxicity and blank values than conventional Pb fire assay. Complete matrix match for the CRMs standards and real geochemical samples were obtained by the modified empirical coefficient method. In order to reduce analysis error and the instability of LA-ICP-MS test parameters, ^109^Ag was selected as internal standard isotope and internal standard calibration strategy was used. Due to the advantages of obtained by solid sample pretreatment and analysis, the sample throughput was improved and interference of polyatomic molecular ions was decreased. The established method was successfully applied to determination of Pt and Pd in real geochemical samples, and the determined values were in good agreement with the results of traditional Pb-FA GF-AAS analysis.

## Supplementary Information

Supplementary Information.
