# Supplementary material for: Bismuth fire assay preconcentration and empirical coefficient LA-ICP-MS for the determination of ultra-trace Pt and Pd in geochemical samples
Source: Sci Rep. 2022 Jul 7;12:11555. doi: 10.1038/s41598-022-15881-5 (PMC9262985; doi:10.1038/s41598-022-15881-5)
Supplement: Supplementary file 1 — Supplementary Information. [file 41598_2022_15881_MOESM1_ESM.docx]

**Bismuth fire assay preconcentration and empirical coefficient LA-ICP-MS for the determination of ultra-trace Pt and Pd in geochemical samples**

Wenshan Ni ^1 a,b,c^ Xiangju Mao ^1 a,b,c^ Mingxing Yao^* a,b,c^ Xiaorui Guo^a,b,c^

Qiliang Sun ^a,b,c^ Xiaofei Gao ^a,b,c^ Hongli Zhang ^a,b,c^

*(a. Zhengzhou Institute of Multipurpose Utilization of Mineral Resources, CAGS,* *Zhengzhou 450006, P. R. China; b. China National Engineering Research Center for utilization of Industrial Minerals, Zhengzhou 450006, P. R. China; c. Key Laboratory of Evaluation and Multipurpose Utilization of Polymetallic Ores Ministry of Natural Resources, Zhengzhou 450006, P. R. China)*

___________________________

^1^ W.S. Ni and X.J. Mao contributed equally to this work.

*Corresponding author, fax: 86-371-68632057, email: mingxingyao_jczx @163.com, address: 328 Longhai west Road, 450006, Zhengzhou, P. R. China

**Table S1** The monitored isotopes of Pt, Pd and Ag

| Element | Isotope | Abundance /% | Isobar |
| --- | --- | --- | --- |
| Pt | ^190^Pt | 0.01 | ^190^Os |
|  | ^192^Pt | 0.79 | ^192^Os |
|  | ^194^Pt | 32.90 | - |
|  | ^195^Pt | 33.80 | - |
|  | ^196^Pt | 25.30 | ^196^Hg |
|  | ^198^Pt | 7.20 | ^198^Hg |
| Pd | ^102^Pd | 1.02 | ^102^Ru |
|  | ^104^Pd | 11.14 | ^104^Ru |
|  | ^105^Pd | 22.33 | - |
|  | ^106^Pd | 27.33 | ^106^Cd |
|  | ^108^Pd | 26.46 | ^108^Cd |
|  | ^110^Pd | 11.72 | ^110^Cd |
| Ag | ^107^ Ag | 51.84 | - |
|  | ^109^ Ag | 48.16 | - |

**Table S2** Possible mass spectral interferences from polyatomic molecular ions on ^195^Pt, ^105^Pd and ^109^Ag.

| Isotope | mass spectral interferences from polyatomic molecular ions |
| --- | --- |
| ^195^Pt | ^14^N^181^Ta, ^1^H^194^Pt, ^16^O^1^H^178^Hf, ^40^Ar^155^Gd, ^12^C^183^W, ^16^O^179^Hf, ^36^Ar^159^Tb, ^13^C^182^W, ^15^N^180^Hf |
| ^105^Pd | ^16^O^89^Y, ^12^C^93^Nb, ^16^O^1^H^88^Sr, ^40^Ar^65^Cu, ^1^H^104^Ru, ^14^N^91^Zr, ^1^H^104^Pd, ^36^Ar^69^Ga, ^13^C^92^Zr, ^15^N^90^Zr, ^13^C^92^Mo |
| ^109^Ag | ^16^O^93^Nb, ^40^Ar^69^Ga, ^1^H^108^Pd, ^16^O^1^H^92^Zr, ^16^O^1^H^92^Mo, ^14^N^95^Mo, ^12^C^97^Mo, ^1^H^108^Cd, ^13^C^96^Mo, ^18^O^1^H^90^Zr |

**Fig. S1** LA-ICP-MS ^105^Pd, ^195^Pt and ^107^Ag signal intensities obtained for the matrix-matched Ag slices. A. blank sample, B. certified sample GBW07293.

**Fig. S2** The calibration curves of ^195^Pt and ^105^Pd by the proposed Bi-FA LA-ICP-MS method.
